# Revisiting the Role of Ser982 Phosphorylation in Stoichiometry Shift of the Electrogenic Na^+^/*q*HCO_3_^−^ Cotransporter NBCe1

**DOI:** 10.3390/ijms222312817

**Published:** 2021-11-26

**Authors:** Thamer A. Alsufayan, Evan J. Myers, Bianca N. Quade, Clayton T. Brady, Aniko Marshall, Nayem Haque, Michael E. Duffey, Mark D. Parker

**Affiliations:** 1 Department of Physiology and Biophysics, University at Buffalo, State University of New York, Buffalo, NY 14203, USA; thamerab@buffalo.edu (T.A.A.); ejmyers@buffalo.edu (E.J.M.); biancaqu@buffalo.edu (B.N.Q.); cbrady@buffalo.edu (C.T.B.); anikomar@buffalo.edu (A.M.); nayemhaq@buffalo.edu (N.H.); duffey@buffalo.edu (M.E.D.); 2 Department of Ophthalmology, Jacobs School of Medicine and Biomedical Sciences, University at Buffalo, State University of New York, Buffalo, NY 14203, USA

**Keywords:** acid-base, epithelium, kidney, colon, ion transport

## Abstract

In most cell types and heterologous expression systems, the electrogenic sodium-bicarbonate cotransporter NBCe1 operates with a 1Na^+^–2HCO_3_^−^ stoichiometry that, given typical transmembrane electrochemical gradients, promotes Na+ and HCO_3_^−^ influx. However, NBCe1 in the kidney mediates HCO_3_^−^ efflux (HCO_3_^−^ reabsorption), a direction that has been predicted to be favored only if NBCe1 operates with a 1:3 stoichiometry. The phosphorylation state of Ser982 in the cytosolic carboxy-terminal domain of NBCe1 has been reported to be a key determinant of the transporter stoichiometry, with non-phosphorylated Ser982 favoring a 1:3 stoichiometry. Conversely, phosphoproteomic data from renal cortical preparations have revealed the presence of NBCe1 peptides including phosphoserine982 (pSer982) and/or pSer985 although it was not known what proportion of NBCe1 molecules were phosphorylated. In the present study, we report the generation, characterization, and application of a novel phosphospecific antibody raised against NBCe1/pSer982 and show that, contrary to expectations, Ser982 is more prevalently phosphorylated in murine kidneys (in which NBCe1 mediates HCO_3_^−^ efflux) than in murine colons (in which NBCe1 mediates HCO_3_^−^ influx). Using phosphomimetic mutants of murine NBCe1 expressed in *Xenopus* oocytes, we found no evidence that the phosphorylation state of Ser982 or Ser985 alone influences the transport stoichiometry or conductance. Furthermore, we found that the phosphorylation of NBCe1/Ser982 is enhanced in murine kidneys following a 24 h induction of metabolic acidosis. We conclude that the phosphorylation status of Ser982 is not a key determinant of NBCe1 stoichiometry but correlates with presumed NBCe1 activity.

## 1. Introduction

The electrogenic Na^+^ bicarbonate cotransporter NBCe1 is a member of the SLC4 family of predominantly HCO_3_^−^ transporting proteins [1,2]. NBCe1 is encoded by the *SLC4A4* gene that produces five gene products, from NBCe1-A to NBCe1-E [3]. In the present manuscript, we confined our interest to the two major variants NBCe1-A and NBCe1-B. NBCe1-A is expressed in the basolateral membrane of the renal proximal tubule epithelia and has a unique 43-amino acid (aa) N-terminal appendage known as the autostimulatory domain (ASD) that distinguishes its sequence from that of NBCe1-B. The ASD confers a constitutively large HCO_3_^−^ conductance, supporting the requirement of the proximal tubule to supply large quantities of HCO_3_^−^ to the plasma in the renal peritubular capillaries [4,5]. NBCe1-B, on the other hand, is expressed in a diverse array of secretory epithelia and non-epithelial cells. NBCe1-B differs from NBCe1-A in the inclusion of an 85-aa N-terminal appendage known as the autoinhibitory domain (AID) in place of the ASD, which confers a relatively lower constitutive HCO_3_^−^-conducting activity to the protein [4]. The AID of NBCe1-B is also a key site of interaction with the secretagogue-activated regulatory binding partner IRBIT (with inositol 1,4,5-trisphosphate receptor binding protein released with inositol 1,4,5-trisphosphate) [6]. IRBIT relieves the autoinhibition of NBCe1-B and promotes a large increase in HCO_3_^−^ conductivity, supporting the requirement of secretory organs such as the colon to increase HCO_3_^−^ secretion on demand [7,8].

**Figure 1:**
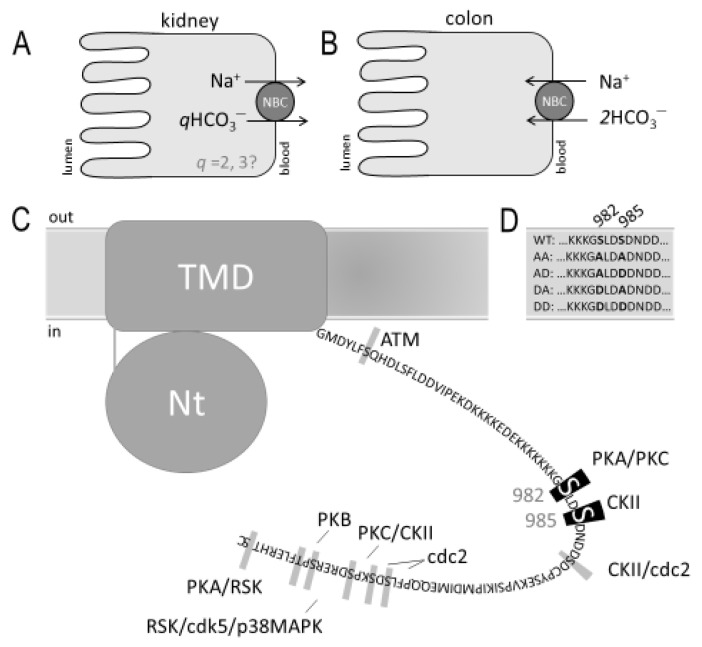
Predicted phosphorylation sites in the cytoplasmic C-terminus of NBCe1. (**A**) The mode of action of NBCe1-A in the basolateral membranes of renal proximal tubule epithelia. HCO_3_^−^ is generated in the cytosol by ammoniagenesis and the action of carbonic anhydrase II; (**B**) The mode of action of NBCe1-B in the basolateral membranes of colonic crypt epithelia. The process of HCO_3_^−^ secretion into the lumen is completed via numerous apical anion transporters and channels [13]; (**C**) Graphic representation of NBCe1-A showing the cytoplasmic amino (Nt) and carboxy (Ct) terminal domains in relation to the transmembrane domain (TMD). Ct is not shown larger than scale in order to present the primary sequence. Highlighted residues were predicted to be phosphorylated (probability > 0.5) by the NetPhos 3.1 server (http://www.cbs.dtu.dk/services/NetPhos/, accessed on 24 February 2021). Black highlighted residues 982 and 985 were demonstrated to be phosphorylated by a phosphoproteomic study of rat kidney cortices [14]. ATM = ATM kinase. PKA, PKB, PKC = protein kinases A, B, and C. cdc2 = cyclin-dependent kinase 1. cdk5 = cyclin-dependent kinase 5. CKII = casein kinase II. p38MAPK = p38 mitogen-activated kinases. RSK = ribosomal S6 kinases; (**D**) The sequence of mutant clones studied in the present work.

The means by which NBCe1-A mediates HCO_3_^−^ reabsorption (i.e., HCO_3_^−^ efflux from the cell, Figure 1A) in the kidney versus the means by which NBCe1-B can support HCO_3_^−^ secretion (i.e., HCO_3_^−^ influx into the cell across the basolateral membrane, Figure 1B) in the colon or the cornea has been the source of much discussion and is not yet fully understood [1]. The default cotransport stoichiometry (*q*) of NBCe1 heterologously expressed in mammalian kidney cell lines [9] and *Xenopus* oocytes [10] is 1Na^+^:2HCO_3_^−^ (i.e., *q* = 2), which favors the influx of Na^+^ and HCO_3_^−^, given the typical basolateral transmembrane electrochemical gradients. In order for NBCe1 to mediate Na^+^/HCO_3_^−^ efflux, the value of *q* has been predicted to be in excess of two [11]. Therefore, in many texts, NBCe1 in the proximal tubule epithelia has been depicted as mediating HCO_3_^−^ reabsorption (i.e., efflux from the cell) with a stoichiometry of 1Na^+^:3HCO_3_^−^. The molecular basis for this difference is neither related to the N-terminal primary sequence differences between NBCe1-A and NBCe1-B, nor in the direction of the electrochemical driving force; the prevailing hypothesis is that NBCe1 is capable of changing its transport stoichiometry in response to yet-to-be identified cell-specific factors [12].

One mechanism by which NBCe1 might change stoichiometry was suggested by a study that implicated the dephosphorylation of a serine residue (Ser982) in the C-terminus of NBCe1-A (Figure 1C: Ser1026 in the C-terminus of NBCe1-B, but for simplicity hereafter, we refer only to “Ser982”, independent of isoform) as being responsible for a shift from *q* = 2 (as proposed in the colon) to *q* = 3 (as proposed in the kidney) [15]. However, a subsequent phosphoproteomic study in rats revealed that NBCe1 fragments, in which Ser982 is phosphorylated, are among the most abundant phosphopeptides detected in renal cortical membrane preparations [14,16]; this observation is seemingly at odds with the proposal that it is NBCe1 with a non-phosphorylated Ser982 that supports renal HCO_3_^−^ reabsorption. In the present manuscript, we sought to clarify the role of Ser982 phosphorylation. First, we used a novel phosphospecific antibody that specifically detects NBCe1 phosphorylated at Ser982, to probe the relative phosphorylation status of NBCe1 in the kidneys and the colons of mice under normal conditions. Second, we used two-electrode voltage-clamp circuitry to examine the stoichiometry of (de)phosphomimetic mutants of NBCe1 expressed in *Xenopus* oocytes. Thirdly, we used our novel antibody to examine the phosphorylation status of Ser982 in murine kidneys following metabolic acidosis, a stimulator of NBCe1 activity. Taken together, our findings do not support the hypothesis that dephosphorylation of Ser982 alone would result in a transition to a 1Na^+^:3HCO_3_^−^ stoichiometry. However, they do suggest that the phosphorylation status of Ser982 was dynamically regulated in a manner that correlated with NBCe1 activity.

## 2. Results

### 2.1. Validating the Anti-pSer982 Antibody

**Figure 2:**
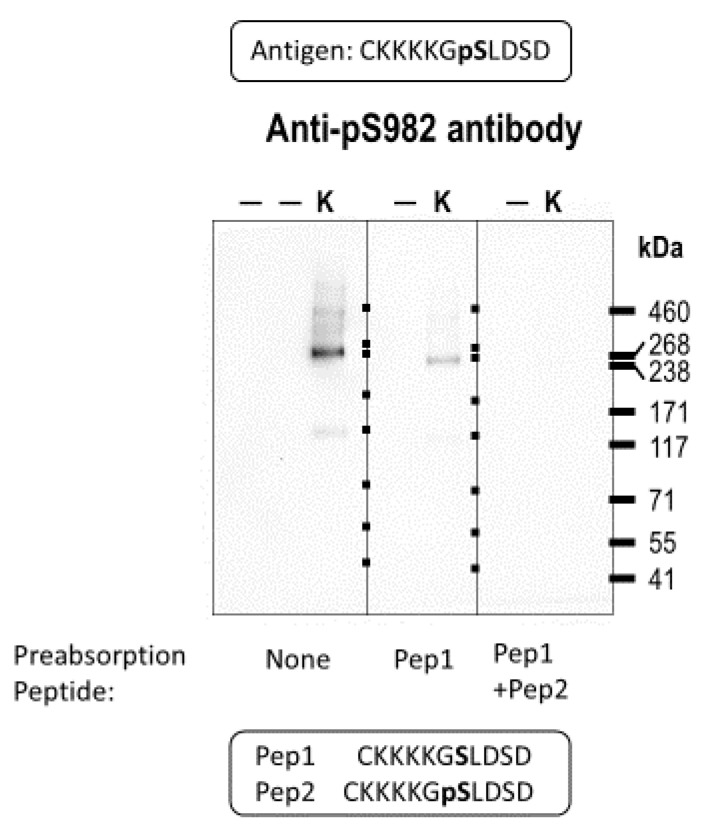
Validation of the pSer982 antibody. Western blot showing anti-pS982 immunoreactivity in murine kidney protein lysates (K = kidney lysate containing lane; — = blank lane). For the middle and right blots, the antibody was preabsorbed with peptides representing the non-phosphorylated version of the epitope (Pep1) with and without a peptide representing the phosphorylated epitope (Pep2).

Figure 2 shows the results of this experiment, in which 2 µg of murine kidney lysate was loaded onto a gel in triplicate and transferred onto a PVDF membrane that was cut into three sections for Western blotting. All three sections were probed with the anti-pSer982 antibody, either with or without pre-absorbing peptide, and the probed blots were exposed for imaging for the same length of time. The first section (Figure 2, left panel) was probed with anti-pSer982 alone and revealed immunoreactive bands at ~120 and ~240 kDa, which were consistent with monomeric and dimeric NBCe1 proteins. The second section was probed with anti-pSer982 antibody that had been preabsorbed for 30 min with 16 µg of a non-phosphorylated version of the antigen (Pep1), and it revealed the same immunoreactive bands, although less intense (Figure 2, middle panel). The third section was probed with anti-pSer982 antibody that had been preabsorbed for 30 min with 8 µg of Pep1 as well as 8 µg of the phosphorylated antigen (Pep2); no immunoreactive bands were detected (Figure 2, right panel). (Note: Because the anti-pSer982 antibody was supplied as a 0.8 mg/mL stock, each blot would be exposed to 8 µg of antibody (1:1000 dilution in 10 mL of blocking buffer). Therefore, given that the peptides have a considerably lower molecular weight than the antibody, we considered 8 µg of each peptide to be a suitable molar excess for immunodepletion.) This result was repeated in at least six independent trials. Therefore, the immunoreactive band in the middle panel represents the NBCe1 protein phosphorylated at Ser982, as its immunoreactivity was specifically competed away by the phosphorylated peptide Pep2. Hereafter, each use of the anti-pSer982 antibody followed preincubation with 16 µg Pep1/10 mL blocking buffer. Anti-pSer982 immunoreactivity was also competed away by a peptide, in which both Ser982 and Ser985 had been phosphorylated (Supplemental Figure S1); therefore, we assumed that the immunoreactivity of the anti-pSer982 antibody against NBCe1 was independent of the phosphorylation state of Ser985.

### 2.2. The Relative Phosphorylation Status of NBCe1/Ser982 in Murine Kidney versus Colon

**Figure 3:**
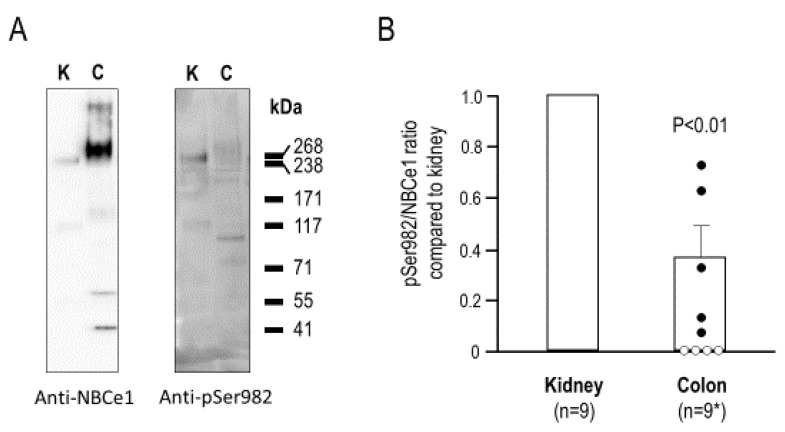
Probing the pSer982 status of kidney and colon NBCe1. (**A**) Western blot showing anti-NBCe1 immunoreactivity and anti-pSer982 immunoreactivity in protein lysates from murine kidney (2 µg/lane) or colon (10 µg/lane); (**B**) Quantification of the ratio of pSer982/NBCe1 in colon versus kidney. Open circles represented null data points due to undetectable pSer982 immunoreactivity in colon and were excluded from the statistical analysis shown.

Figure 3A shows a representative experiment in which we probed NBCe1 immunoreactivity in individual protein preparations from the kidneys and proximal colons of nine mice. The left panel of Figure 3A shows the expected immunoreactive bands at ~120 and ~240 kDa, with the bands from colon exhibiting a higher molecular weight consistent with the slightly larger size of colon NBCe1 (variant NBCe1-B), as compared to kidney NBCe1 (variant NBCe1-A). From these blots, we determined the ratio of colon to kidney anti-SLC4A4 immunoreactivity as well as the corresponding ratio of kidney to colon anti-pSer982 immunoreactivity. For five of the nine mice, we were able to determine the fractional difference between these ratios (i.e., the bar and the filled circles in Figure 3B.) A value less than one indicated that fewer NBCe1 molecules were phosphorylated at Ser982 in the colon than in the kidney. The open circles in Figure 3B were not included in the average and represented the four out of the nine mice for which, despite robust anti-pSer982 immunoreactivity in the kidneys, anti-pSer982 immunoreactivities in the colons were indistinguishable from the background, and thus, a ratio could not be reliably determined. These zero values were omitted from the statistical analysis; even without these zero values, we found that the NBCe1-B in the proximal colon was significantly less phosphorylated at this residue than the NBCe1-A in the kidneys of the mice (two-tailed, paired *t*-test.)

### 2.3. The Electrophysiological Properties of Double (de)Phosphomimetic Mutants of NBCe1-A and NBCe1-B in Xenopus Oocytes

**Figure 4:**
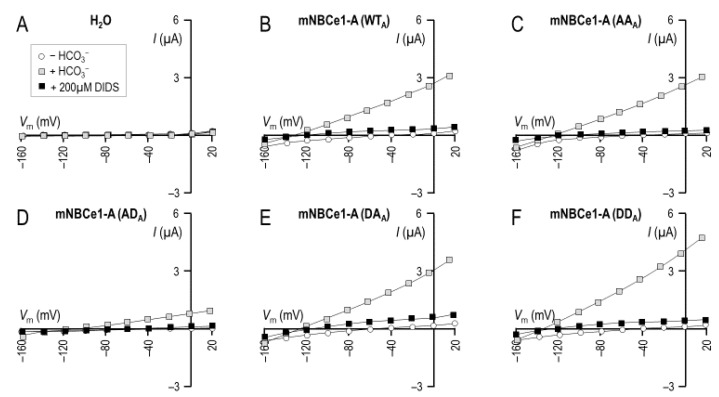
I–V relationships for oocyte expressing (de)phosphomimetic NBCe1-A clones. Each panel shows data gathered from a single representative oocyte injected with either (**A**) H_2_O, (**B**) cRNA-encoded wild-type NBCe1-A, or (**C**–**F**) cRNA encoded with one of five (de)phosphomimetic mutants. Open circles are I–V data gathered from oocytes during perfusion with our CO_2_/HCO_3_^−^-free solution, gray squares are I–V data gathered during perfusion with our CO_2_/HCO_3_^−^-containing solution, and black squares are I–V data gathered during perfusion with our CO_2_/HCO_3_^−^-containing solution plus 200 µM DIDS.

Considering the possibility that the phosphorylation status of nearby residue Ser985 had the potential to influence Ser982-related phenomena, we mutated Ser982 and Ser985 together to generate a set of NBCe1-A-EGFP mutants, in which the status of both sites was (de)phosphomimicked. These mutants were NBCe1-A-EGFP/S982A/S985A (AA_A_), NBCe1-A-EGFP/S982A/S985D (AD_A_), NBCe1-A-EGFP/S982D/S985A (DA_A_), and NBCe1-A-EGFP/S982D/S985D (DD_A_). We expressed each clone from cRNA in *Xenopus* oocytes and determined *E*_rev_ for each using a two-electrode voltage clamp. Figure 4A shows a representative current–voltage (I–V) relationship gathered from an H_2_O-injected oocyte as it was sequentially exposed to either a HCO_3_^−^-free solution (open circles), a HCO_3_^−^-containing solution (gray square), or the same HCO_3_^−^-containing solution that included 200 µM DIDS (black squares).

**Figure 5:**
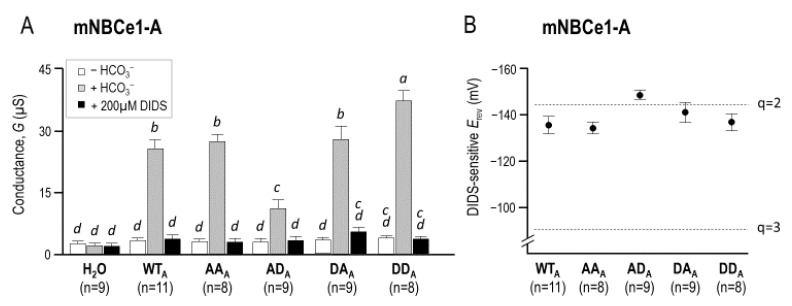
The conductance and stoichiometry of (de)phosphomimetic NBCe1-A clones in *Xenopus* oocytes. (**A**) The membrane conductance of a larger number of oocytes calculated from data, such as those shown in Figure 4. Letters above bars indicate statistically indistinguishable groups (i.e., groups with different letters were significantly different from each other); (**B**) The reversal potentials of wild-type NBCe1-A-EGFP and the four (de)phosphomimetic mutants plotted against the reversal potentials that correspond to predicted transport stoichiometries of 1Na^+^–3HCO_3_^−^ (*q* = 3) and 1Na^+^–2HCO_3_^−^ (*q* = 2).

These maneuvers elicited no significant alterations in membrane conductance (*G*_m_), as shown for a larger number of H_2_O-injected cells in Figure 5A. Figure 4B shows a representative I–V relationship gathered from a wild-type (WT_A_) NBCe1-A-EGFP-injected oocyte as it was sequentially exposed to the same solutions. The exposure to HCO_3_^−^ elicited a large increase in *G*_m_ and a hyperpolarization of the membrane potential (*V*_m_) that is characteristic of NBCe1-expressing oocytes (e.g., [17]). Furthermore, the subsequent application of DIDS caused *G*_m_ to fall. Figure 4C–F show a similar response to these maneuvers from cells expressing NBCe1-mutants AA_A_, AD_A_, DA_A_, and DD_A_. These responses are summarized for a larger number of cells in Figure 5A. Analysis by general linear model with post hoc analysis revealed that all cells expressing NBCe1 exhibited a DIDS-sensitive HCO_3_^−^ conductance in their membranes. The intersection of I–V relationships plotted in the presence versus the absence of DIDS in HCO_3_^−^-containing solution reports the *E*_rev_ of the transport process. These data are shown in Figure 5B, alongside the predicted *E*_rev_ values (dashed lines) for NBCe1 working with a 1Na–2HCO_3_ cotransport stoichiometry (*q* = 2) or a 1Na–3HCO_3_ cotransport stoichiometry (*q* = 3). Our data were most consistent with a 1:2 stoichiometry for all NBCe1 clones tested.

**Figure 6:**
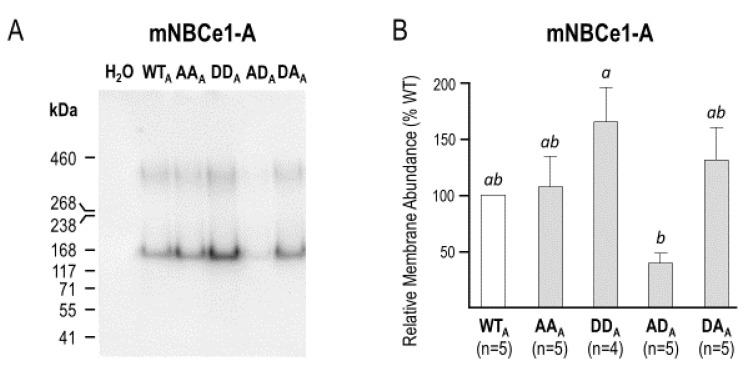
The relative membrane abundance of (de)phosphomimetic NBCe1-A clones in *Xenopus* oocytes. (**A**) Western blot showing EGFP immunoreactivity in biotinylated protein fractions (i.e., cell-surface-expressed) NBCe1-A-EGFP from *Xenopus* oocytes; (**B**) Quantification of the relative plasma-membrane expression of (de)phosphomimetic mutants of NBCe1-A, as compared to wild-type. Letters above bars indicate statistically indistinguishable groups (i.e., groups with different letter were significantly different from each other).

The statistical analysis in Figure 5A suggested significant differences in *G*_m_ among the mutants, with the cell membranes expressing AD_A_ being significantly less conductive than those expressing DD_A_. In order to determine whether this represented an alteration in the intrinsic NBCe1 activity, we performed biotinylation on five sets of oocytes expressing these clones. Figure 6A shows a representative Western blot of biotinylated (i.e., plasma-membrane-resident) NBCe1 protein probed with an anti-EGFP antibody. Note that the immunoreactive bands were larger than those found in native NBCe1 in Figure 2 and Figure 3. This is due to the presence of a C-terminal-EGFP tag. The densities of the immunoreactive bands were quantified and averaged for each clone, as shown in Figure 6B. One-way ANOVA indicated that, as with *G*_m_ in Figure 5A, plasma membrane abundance was greater for DD_A_ than AD_A_. Taken altogether, these results suggested that the phosphomimetic mutations did not substantially affect the per-molecule transport activity of NBCe1-A.

**Figure 7:**
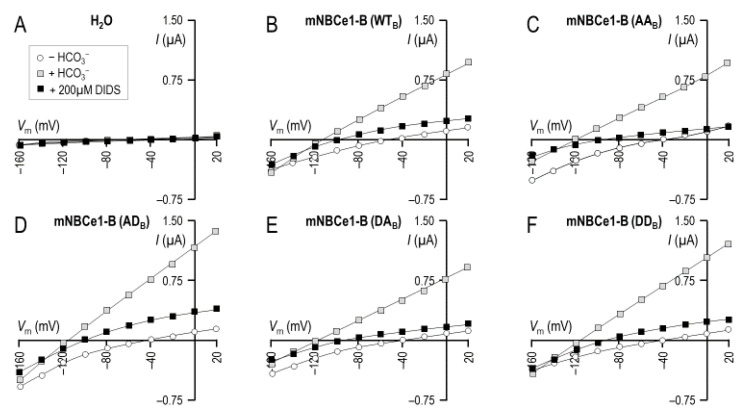
I–V relationships for oocyte expressing (de)phosphomimetic NBCe1-B clones. Each panel shows data gathered from a single representative oocyte injected with either (**A**) H_2_O, (**B**) cRNA-encoded wild-type NBCe1-B-EGFP, or (**C**–**F**) one of five cRNA-encoded (de)phosphomimetic mutants. Open circles are I–V data gathered from oocytes during perfusion with our CO_2_/HCO_3_^−^-free solution, gray squares are I–V data gathered during perfusion with our CO_2_/HCO_3_^−^-containing solution, and black squares are I–V data gathered during perfusion with our CO_2_/HCO_3_^−^-containing solution plus 200 µM DIDS.

**Figure 8:**
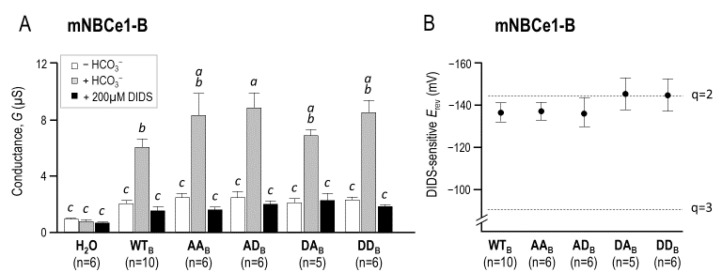
The conductance and stoichiometry of (de)phoshomimetic NBCe1-B clones in *Xenopus* oocytes. (**A**) The membrane conductance of a larger number of oocytes calculated from data, such as those show in Figure 7. Letters above bars indicate statistically indistinguishable groups (i.e., groups with different letter were significantly different from each other); (**B**) The reversal potentials of wild-type NBCe1-B and the four (de)phosphomimetic mutants plotted against the reversal potentials that correspond to predicted transport stoichiometries of 1Na^+^–3HCO_3_^−^ (*q* = 3) and 1Na^+^–2HCO_3_^−^ (*q* = 2).

Figure 7 and Figure 8 show an equivalent data to the data presented in Figure 4 and Figure 5, except for the (de)phosphomimetic mutants made on a background of NBCe1-B-EGFP (WT_B_) instead of NBCe1-A-EGFP. Figure 8A shows that the mutant-expressing oocytes exhibited a similar *G*_m_ to the WT_B_-expressing oocytes while Figure 8B shows that the *E*_rev_ for all clones was most consistent with a transport stoichiometry of 1Na^+^–2HCO_3_^−^.

### 2.4. The Relative Phosphorylation Status of NBCe1/Ser982 in Murine Kidneys after 24 h Metabolic Acidosis

The greater extent of the phosphorylation at Ser982 in the kidney NBCe1 than in the colon NBCe1 is consistent with the hypothesis suggesting phosphorylation at this residue would correlate with NBCe1 activity. Therefore, we hypothesized that metabolic acidosis, a condition that requires an increase in renal HCO_3_^−^ generation, could increase phosphorylation at Ser982 in renal NBCe1. Our study group included seven control mice and eight NH_4_Cl-dosed mice. We performed blood-gas analysis on five of our control mice and four of the NH_4_Cl-dosed mice to confirm induction of metabolic acidosis ([HCO_3_^−^] = 25 ± 1 mM for control, 14 ± 1 mM for NH_4_Cl-dosed, *p* < 0.01; unpaired, one-tailed *t*-test.)

**Figure 9:**
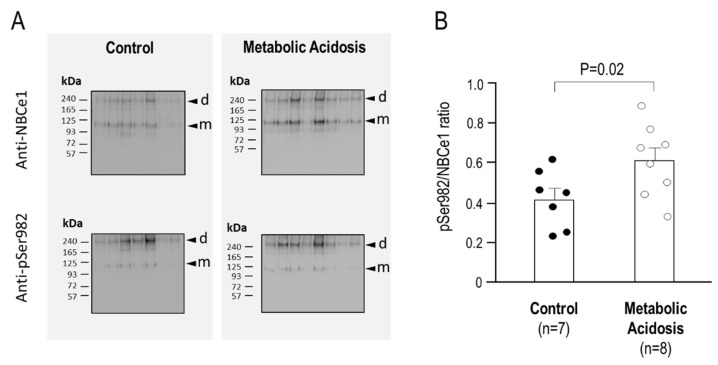
The influence of 24 h metabolic acidosis on the pSer982 status of renal NBCe1 protein. (**A**) Western blots showing anti-NBCe1 and anti-pSer982 immunoreactivity (dimer (d), and monomer (m)) in mice that had ad libitum access to drinking water containing 0.5% sucrose (control) or 0.5% sucrose plus 0.28 M NH_4_Cl (metabolic acidosis); (**B**) Quantification of the ratio of pSer982/NBCe1 in control versus metabolic acidotic mice. Equal protein loading and transfer among blots was confirmed using MemCode reversible (total) protein stain (see Supplemental Figure S3).

Kidney preparations of all mice were blotted in parallel with either the anti-NBCe1 antibody or the anti-pSer982 antibody. The two blots for each experimental group (control vs. acidosis) were imaged together, which is to say that the two blots on the left side of Figure 9A were cropped from a single image as were the two blots on the right side of Figure 9A. The ratio of pSer982/SLC4A4 immunoreactivity was calculated for the control mice and for the acidotic mice; these data are plotted in Figure 9B, revealing significantly greater phosphorylation at Ser982 of NBCe1 in the acidotic mice: an average increase of 46% when the bands corresponding to SDS-resistant dimers and monomers were quantified together (*p* = 0.02; one-tailed, unpaired *t*-test, per Figure 9B), an average increase of 71% (*p* < 0.01) when dimers alone were quantified, and an average increase of 29% (n.s.) when monomers alone were quantified.

## 3. Discussion

Several solute-carrier families include members with distinct transport stoichiometries (e.g., the Na^+^/glucose cotransporters SGLT1 and SGLT2, and the Na^+^/phosphate cotransporters Npt2 and NaPi-IIc), but the concept that a single transporter could have variable stoichiometry is uncommon. Much evidence has suggested that that a single transporter such as NBCe1, or the related NBCe2 (SLC4A5) in the choroid plexus epithelia [18], may adopt a Na^+^–HCO_3_^−^ stoichiometry of 1:3 to overcome the presumed electrochemical gradients and mediate HCO_3_^−^ efflux (reviewed in [1]). Additionally, calculations relating to NBCe1 in astrocytes have suggested that electrochemical gradients could support a HCO_3_^−^-efflux mode even with a 1:2 transport stoichiometry [19,20].

Previous studies on the mechanism by which such a stoichiometric switch could occur for NBCe1 have consistently found that the phosphorylation of Ser982 could cause a stoichiometry transition from 1:3 to 1:2 and, thereby, induce a transition from a HCO_3_^−^-efflux mode to a HCO_3_^−^-influx mode [15,21]. Therefore, it would be expected that a smaller fraction of NBCe1 molecules ought to be phosphorylated at Ser982 in the kidney than, for example, in the colon. With that in mind, it was surprising to discover that the phosphoproteomic data of rat kidney cortices found by Feric et al. [14] showed that a phosphopeptide corresponding to a fragment of NBCe1, in which both Ser982 and Ser985 were phosphorylated, was the 13th most abundant out of the 743 phosphopeptide signatures detected. Adding to this total, although less individually abundant, were the NBCe1 peptides that were phosphorylated at only Ser982 or Ser985. At face value, these data appeared to contradict the findings from earlier studies. However, these data were not necessarily incompatible, as the phosphoproteomic results did not detail what proportion of NBCe1 molecules were phosphorylated at Ser982 in the kidney; the phosphopeptides may have been abundant because NBCe1 is abundant in the proximal tubule. Moreover, comparable data from the colon were not available. In order to reconcile these observations, we generated and validated what we believe to be the first phosphospecific antibody against an Na^+^-coupled HCO_3_^−^ transporter. The results of the kidney and colon blots with our novel anti-pSer982 antibody demonstrated, for the first time, the unexpected observation that a greater proportion of NBCe1 is phosphorylated at Ser982 in the kidney than in the colon (Figure 3). These results suggest a need for reexamination of the conclusions of earlier structure–function studies.

It is first worth noting that one earlier study had concluded that NBCe1 could indeed perform HCO_3_^−^ reabsorption even with a 1:2 stoichiometry in the proximal tubule [22]. In other words, the stoichiometry of NBCe1 may not need or be able to change stoichiometry; instead, our knowledge of the electrochemical driving forces acting across the basolateral membranes of these cells in vivo may be incomplete. However, if we assume that a pSer982-linked difference in stoichiometry between NBCe1 in the kidney and colon is critical to its physiological function, then we must also assume that the majority of the NBCe1 protein in the kidney (or colon) must adopt the appropriate phosphorylation state for the population to mediate net HCO_3_^−^ efflux (or influx). In other words, according to the original hypothesis, the majority of NBCe1 in the kidney must not be phosphorylated at Ser982 and the majority of NBCe1 in the colon must be phosphorylated at Ser982. Our data could not be used to determine an absolute value for the proportion of NBCe1 that was phosphorylated in each organ because we do not know the relative affinities of the SLC4A4 and pSer982 antibodies to their targets. However, we loaded an excess of colon NBCe1, as compared to kidney NBCe1, on our gels (Figure 3A) and, for the most part, could barely detect pS982 immunoreactivity in the colon preparation (Figure 3B) despite the robust renal pS982 immunoreactivity on the same blots. Therefore, our findings indicated that colonic NBCe1 was predominantly unphosphorylated at Ser982. Regarding NBCe1 in the kidney, our data showed that more NBCe1 was phosphorylated at Ser982 in the kidney than in the colon, but we could not determine from our data alone whether this represented a small or large fraction of the total kidney NBCe1 population. However, given the robust representation of NBCe1 pSer982 phosphopeptides in the earlier phosphoproteomic study, we suspect that it will not be a trivial fraction. In addition, the fact that acidosis substantially increased pSer982 abundance in the kidney by ~50% on average may provide an approximate upper limit of 66% to the estimated fraction of NBCe1 that was phosphorylated at Ser982 under normal conditions. Nonetheless, these data did not discount the possibility—consistent with the earlier model—that the majority of NBCe1 is not phosphorylated at Ser982 in the kidney, and that it is this majority population of non-pSer982 NBCe1, in combination with the influence of unidentified kidney-specific factors, that allows for the adoption of the 1:3 efflux mode. However, this scenario is unlikely since we observed an ~50% increase in the phosphorylation of Ser982 during acidosis (Figure 9). This change would, according to the previous model, encourage a counterintuitive shift towards the 1:2 influx mode when acidosis should instead cause an compensatory increase in proximal tubular HCO_3_^−^ efflux [23,24]. Overall, our data are consistent with a model in which the phosphorylation of Ser982 in a given NBCe1 population (1) correlates with presumed NBCe1 activity (i.e., was proportionally greater in the kidney versus the colon and increased in the kidney during metabolic acidosis), but (2) if such a mechanism exists, would correlate with the presumed 1:3 stoichiometry rather than the 1:2 stoichiometry. The reason for this disparity is not obvious although earlier studies relied on indirect methods of assessing the phosphorylation status of Ser982 and, as has been mentioned elsewhere, the data in support of stoichiometry shifts in various conditions may have been influenced by the underappreciated quirks of the assay methods and the undue influence of the background currents [1,25].

In order to directly study the relationship between NBCe1 stoichiometry and Ser982 phosphorylation status, we assayed a set of phosphomimetic NBCe1-A and NBCe1-B mutants in *Xenopus* oocytes. Both wild-type clones exhibited the 1:2 stoichiometry (Figure 5 and Figure 8), which was consistent with previous observations of NBCe1-A behavior when heterologously expressed in oocytes [10] (we believe that our study may the first formal demonstration of the 1:2 stoichiometry of NBCe1-B in oocytes) and with the notion that stoichiometry is not determined by the differences in the primary sequence between NBCe1-A and NBCe1-B [12]. One mechanism by which phosphorylation at Ser982 has been proposed to engender a stoichiometry shift from 1:2 to 1:3 is by the disruption of an electrostatic interaction between the ion transporting membrane domain and the cytoplasmic C-terminus of NBCe1, which could uncover a third cryptic HCO_3_^−^ binding site [21]. If, as hypothesized, this effect was purely electrostatic, then it ought to be observable in any cell type. However, we found no evidence that disrupting or phosphomimicking the phosphorylation state of NBCe1-A or NBCe1-B in *Xenopus* oocytes causes a deviation from the typical 1:2 stoichiometry (Figure 5 and Figure 8), contrary to the earlier model that predicted that Ser982A mutants would exhibit a 1:3 stoichiometry.

Setting aside stoichiometry, what did our data suggest about the role of pSer982? The stark contrast in the phosphorylation status of Ser982 between the kidney and the colon and the ability of the pSer982/NBCe1 ratio to be enhanced in acidosis showed that the residue had been deliberately regulated in a manner that appeared to correlate with the requirement for enhanced HCO_3_^−^ flux. Ser982 has been a predicted target for protein kinase A (PKA) as well as protein kinase C (PKC). Earlier hypotheses have focused on the role of PKA, which had been demonstrated to inhibit renal HCO_3_^−^ reabsorption, and therefore, the phosphorylation of Ser982 had been presumed to correlate with a reduction in NBCe1 activity [15]. However, what we have since come to appreciate is that PKC could also phosphorylate Ser982. PKC signaling is a key activator of renal HCO_3_^−^ transport responding to acidosis and angiotensin II [26,27], which would allow for a stimulatory role of phosphorylation of Ser982. PKC-mediated phosphorylation of Ser982 would be consistent with our observation that acidosis increased the pSer982/NBCe1 ratio (Figure 9) although we did not investigate the underlying signaling pathway. Considering the colon, it is known that both of the secretagogues forskolin (that acts through PKA) and carbachol (that acts through PKC) stimulate NBCe1 action [28,29], and thus the demonstration of the increased phosphorylation of Ser982 in the colon by either of these stimuli could add much value to future discussions. We did attempt such experiments in colonic crypt-cell preparations, but the variability in the data—which we attributed to the low level of Ser982 phosphorylation in the colon and the variability in cell viability—hindered our ability to gather a data set with sufficient statistical power. In regard to the correlation between the phosphorylation state of Ser982 and HCO_3_^−^ transport, our oocyte data suggested that the mechanism linking pSer982 with increased HCO_3_^−^ flux in vivo could not be attributed to an increase in the per molecule NBCe1-mediated Na^+^/*q*HCO_3_^−^ flux (Figure 5, Figure 6 and Figure 8). Presumably, the mechanism involves the enhancement of the plasma membrane abundance of NBCe1, either by promoting its forward trafficking from vesicles into the membrane or by increasing its residency time in the plasma membrane. This would be consistent with what is known about the mechanism of activation of NBCe1 in the proximal tubule (an increase in HCO_3_^−^ efflux with no increase in total NBCe1 abundance, as reviewed in [1]) and the colonic epithelia (an increased release of NBCe1 to the plasma membrane [8]). Our oocyte data also suggested that the phosphorylation of Ser982, on the background of a phosphorylated Ser985, could increase the plasma-membrane residency of NBCe1-A although our experiments were not performed with that hypothesis in mind nor did we investigate this phenomenon further. However, preliminary data suggest that all four NBCe1-A mutants were capable of expression to the basolateral plasma membrane of the polarized MDCK (Madin-Darby canine kidney) cells [30].

Analysis of the data from Figure 9B indicated that acidosis had caused a preferential phosphorylation of the SDS-resistant dimers of NBCe1-A, as compared to the monomers. NBCe1 is believed to be predominantly dimeric in vivo [31,32] and the ratio of SDS-resistant dimer vs. monomer (presumably equivalent to SDS-sensitive dimer) evident on the blots is likely to be more reflective of sample processing (e.g., denaturation) than anything biological. However, the result does encourage speculation that there may be a link between phosphorylation and some conformational change that is reflected in the SDS-resistance of the dimers. Whether cause (i.e., phosphorylation contributes to stabilization) or effect (e.g., a fraction of NBCe1–A affects a stable conformation that is more susceptible to phosphorylation), could not be determined from our data.

In summary, our data did not discount the possibility of a stoichiometry shift by NBCe1 but were inconsistent with the previously postulated mechanism by which the phosphorylation of Ser982 alone, and by simple electrostatic means, effects a shift to a 1:2 stoichiometry. Future avenues of investigation may probe the phosphorylation status of Ser985 as well as other consequential phosphorylation sites in the N-terminus that have been highlighted by others [33,34] to further characterize the combinatorial effects of phosphorylation at these sites and mechanisms of action of NBCe1 stimuli in a variety of organs.

## 4. Materials and Methods

### 4.1. cDNA Clones and Mutagenesis

Mouse NBCe1-A and mouse NBCe1-B cDNAs in the pGH19 *Xenopus* expression vector were a kind gift from Liming Chen [3]. In both clones, the NBCe1 cDNA was followed by the 3′UTR of *Xenopus* β-globin and a NotI restriction site that was used to linearize the construct prior to cRNA synthesis. In order to add an in-frame C-terminal EGFP sequence to each clone, we inserted an AgeI restriction site by Quikchange II site-directed mutagenesis (Agilent, Santa Clara, CA, USA) prior to the termination codon of NBCe1. The primers used were forward: 5′-CGCCACACATCATGCtcaccggtTGATAAAATTCCTTTC-3′ and reverse: 5′-GAAAGGAATTTTATCAACCGGTGAGCATGATGTGTGGCG-3′, in which the termination codon is underlined and the introduced sequence is in lowercase letters. The additional “tc” sequence introduced with the AgeI site was required to keep the NBCe1 in frame when the EGFP open reading frame was introduced. The introduced sequence enabled the 3′UTR of each clone to be excised with an AgeI/NotI double digest and replaced by ligation with an equivalent AgeI/NotI-restricted DNA fragment from a donor vector [35] containing EGFP and followed by a termination codon and the same 3′UTR.

Phosphomimetic mutations were introduced into NBCe1-A and NBCe1-B clones using Quikchange II site-directed mutagenesis (Agilent) to produce double mutants for each that we named “AA” (using NBCe1-A numbering: Ser982Ala/Ser985Ala, using NBCe1-B numbering Ser1026Ala/Ser1029Ala), “AD” (Ser982Ala/Ser985Asp or Ser1026Ala/Ser1029Asp), “DA” (Ser982Asp/Ser985Ala or Ser1026Asp/Ser1029Ala), and “DD” (Ser982Asp/Ser985Asp or Ser1026Asp/Ser1029Asp). The sequence of these mutants is depicted in Figure 1D. The forward (FWD) and reverse (RVS) mutagenesis primers used to generate them are listed in Table 1. In the text, a subscript “A” or “B” denotes the template clone (NBCe1-A or NBCe1-B).

### 4.2. cRNA Preparation

A total of 5 µg of each cDNA clone was linearized with NotI and purified using the QIAGEN MinElute PCR purification kit (QIAGEN, Germantown, MD). A total of 1 µg of purified, linearized cDNA was used for in vitro cRNA synthesis with a T7 mMessage mMachine kit (Thermo Fisher Scientific, Waltham, MA). The cRNA was purified using the QIAGEN RNeasy MinElute cleanup kit (QIAGEN).

### 4.3. Oocyte Preparation

Ovaries were removed from 0.2%-tricaine anesthetized *Xenopus laevis* (Xenopus Express, Brooksville, FL, USA) according to the protocol approved by the Institutional Animal Care and Use Committee at the University at Buffalo. Harvested ovaries were dissected into <1 cm^3^ pieces, rinsed in Ca^2+^-free solution, and digested with collagenase to liberate individual oocytes as recently described in [36]. Oocytes were injected either with 25 nL of water or 25 nL of 1 µg/µL cRNA and incubated at 18 °C until use in OR3 medium: 14 g Leibovitz’s L-15 medium powder (Thermo Fisher Scientific, Waltham, MA, USA), 5 mM HEPES, and 20 mL 100× penicillin–streptomycin solution (Corning, Corning, NY, USA); osmolality was adjusted to 200 mOsmol/kg with water and pH adjusted to 7.50 with NaOH.

### 4.4. Electrophysiology

The electrophysiology arrangement was described in [36]. Briefly, oocytes were placed in a recording chamber filled with ND96 solution (in mM: 93.5 NaCl, 2 KCl, 1.8 CaCl_2_, 1 MgCl_2_, and 5 HEPES; pH adjusted to 7.50 with NaOH) and impaled with two KCl-filled borosilicate microelectrodes connected to an OC275 oocyte clamp (Warner Instruments, Hamden, CT, USA) to permit the gathering of current–voltage relationships from the oocyte plasma membrane. The I–V data were digitized and acquired using a Digidata 1550 and Clampex 10.4 software (Molecular Devices LLC, San Jose, CA, USA) while a 725I oocyte bath clamp (Warner Instruments, San Diego, CA, USA) held the bath potential at 0 mV. Once a stable membrane potential had been achieved, I–V relationships were obtained by clamping the cell membrane potential (*V*_m_) at its spontaneous value and then from –160 mV to +20 mV in 20 mV steps for 100 ms, returning to the resting potential for 100 ms in between each step. The bathing solution was exchanged for a CO_2_/HCO_3_^−^-containing solution (in mM: 60.5 NaCl, 2 KCl, 1.8 CaCl_2_, 1 MgCl_2_, and 5 HEPES; pH adjusted to 7.50 with NaOH and 33 NaHCO_3_, then pH readjusted to 7.50 with 5% CO_2_), and once the peak hyperpolarization had been observed, a new I–V relationship was obtained. Finally, the solution was exchanged for a similar solution that contained 200 µM DIDS. The slope conductance for each clone in each solution (*G*_m_) was determined as the slope of the I–V relationship between –20 mV and + 20 mV. The reversal potential (*E*_rev_) for each clone was determined as the point of intersection between I–V relationships gathered in CO_2_/HCO_3_^−^-containing solutions ± DIDS.

### 4.5. Oocyte Membrane Protein Preparation

Plasma-membrane-resident proteins were labeled with biotin using the Pierce Cell Surface Protein Isolation Kit (Thermo Fisher Scientific) with a protocol modified for oocytes. In brief, the supplied biotinylation reagent was dissolved in PBS (osmolality adjusted to 200 mOsm/kg), and then 5mL of the solution was used to incubate each group of ten cells. Following the kit-mandated quenching step, each group of oocytes was manually homogenized in 500 µL of PBS (containing proteinase inhibitors and 1% Triton X100), and insolubilized material was isolated by a 5 min centrifugation at 3000 rpm. The supernatant was applied to a neutravidin column, and the extraction of biotinylated protein proceeded, according to the kit manufacturer’s recommendation. Finally, the biotinylated protein was eluted from the column using 500 µL of 1× SDS loading buffer containing 50 mM DTT. For Western blotting, one-tenth of the eluate was loaded per gel lane (i.e., one-oocyte equivalent).

### 4.6. Mouse Studies and Institutional Approval

For the experiments shown in Figure 2 and Figure 3, we used wild-type C57BL/6J mice that had undergone no experimental intervention. For the experiments shown in Figure 9, we used wild-type C57BL/6J mice that, for 24 h prior to euthanasia, had ad libitum access to drinking water that contained 0.5% sucrose (control) or 0.5% sucrose plus 0.28 M NH_4_Cl (to induce metabolic acidosis (MAc), as described in [37]). All mice were euthanized by CO_2_ overdose followed by cervical dislocation, with the exception of the mice from the MAc study, which were anesthetized with isoflurane and exsanguinated via cardiac puncture to obtain a blood sample. The induction of MAc was confirmed by blood-gas analysis (mixed arterial–venous blood gathered by cardiac puncture) using an epoc blood analysis system (Siemens Medical Solutions, Malvern, PA, USA).

### 4.7. Mouse Kidney and Colon Membrane Protein Preparation

After euthanasia, both kidneys and/or the most proximal third of the colon (~2 cm) were removed and placed into an ice-cold homogenization buffer (in mM: 100 NaCl, 25 HEPES, 1 EDTA, and 250 sucrose; pH adjusted to 7.4 with NaOH) containing phosphatase inhibitors (in mM: 50 NaF and 10 Na_4_P_2_O_7_) and protease inhibitors (Pierce Protease Inhibitor tablets #A32963, Thermo Fisher Scientific, Waltham, MA, USA). Kidneys were homogenized in 1.5 mL Eppendorf tubes containing 1 mL of the buffer using a plastic pestle (#47747-358, VWR, Radnor, PA, USA) and finished off by passage through an 18 G needle, followed by a 21 G needle. Colons, having tougher tissue, were cut into 3 or 4 smaller pieces and homogenized in 2mL of buffer using a D1000 hand-held homogenizer (Benchmark Scientific, Sayreville, NJ, USA), followed by passage through an 18 G and then a 21 G needle. For both organs, homogenization-resistant material was removed by centrifugation at 1075× *g* for 15 min. Insoluble materials (i.e., membrane fragments) were isolated from the supernatant by ultracentrifugation at 41,000 rpm for 45 min using an SW 55Ti swing bucket rotor (Beckman Coulter, Indianapolis, IN, USA). The resulting pellets were resuspended in 500 µL of homogenization buffer, and protein concentration was quantified using a Pierce BCA Protein Assay Kit (Thermo Fisher Scientific). Protein was solubilized to a final concentration between 2–10 µg/20 µL in NuPAGE LDS sample buffer (Thermo Fisher Scientific, Waltham, MA, USA). For Western blotting, 20 µL of each preparation was loaded per lane.

### 4.8. Generation of the Anti-pSer982 Antibody

The anti-pSer982 antibody was generated by Pierce Biotechnology (Rockford, IL) in rabbits using the immunizing peptide C-KKKKG(pS)LDSD (Pep2), in which the N-terminal cysteine was used to conjugate the peptide to keyhole limpet hemocyanin. The antibody was affinity-purified using the same peptide.

### 4.9. Protein Electrophoresis and Western Blotting

Protein extracts were run on NuPAGE 3%–8% Tris-acetate gels alongside 5 µL of BLUelf prestained protein ladder (FroggaBio, Concord, ON, Canada) or HiMark prestained protein standard (Thermo Fisher Scientific). Blank lanes were loaded with 20 µL NuPAGE LDS sample buffer. Protein bands were transferred onto Invitrolon PVDF membranes (Invitrogen, Carlsbad, CA, USA) and stored in blocking buffer (5% milk powder and 0.1% Tween 20 in TBS) overnight. NBCe1 protein was probed using a 1:1000 dilution of rabbit anti-SLC4A4 polyclonal antibody (#E-AB-14348, Elabscience, Houston, TX, USA; see [38] and Supplemental Figure S2 for validation) or 1:1000 of our novel rabbit anti-pSer982 antibody (preabsorbed for 30 min with 16 µg/10 mL blocking buffer of peptide (Pep1); see manuscript results for details of validation). Exceptions were the data in Figure 9, for which we incidentally used a different anti-NBCe1-A/B antibody [39] at a 1:1000 dilution. Immunoreactivity was disclosed using 1:2000 of HRP-conjugated goat-anti-rabbit secondary antibody (MP Biomedicals, Solon, OH, USA) with either Pierce ECL Plus Western Blotting Substrate or SuperSignal West Femto reagent (Thermo Fisher Scientific, Waltham, MA, USA).

### 4.10. Data Analysis and Statistics

Data were reported as means ± standard error of the mean. *E*_rev_ values for a Na^+^/*q*HCO_3_^−^ cotransporter, when *q* = 2 or *q* = 3, were calculated using known extracellular concentrations of Na^+^ (96 mM) and HCO_3_^−^ (33 mM) in our CO_2_/HCO_3_^−^-containing bath solution, and typical values of intracellular Na^+^ (5 mM) and HCO_3_^−^ (9 mM) for a *Xenopus* oocyte expressing NBCe1-A under these conditions [40] were found using the equation:

(1) $$E_{\mathrm{rev}}=\frac{1}{q-1}\frac{RT}{F}\ln\left[\frac{{\left[{\mathrm{Na}}^{+}\right]}_{i}}{{\left[{\mathrm{Na}}^{+}\right]}_{o}}{\left(\frac{{\left[{{\mathrm{HCO}}_{3}}^{-}\right]}_{i}}{{\left[{{\mathrm{HCO}}_{3}}^{-}\right]}_{o}}\right)}^{q}\right]$$

Densitometry was performed on Western blots using Fiji software [41]. Statistical analyses were performed using MiniTab 19, and test details were given with each experiment.

## Figures and Tables

**Table 1:** Mutagenesis primers used to generate (de)phosphomimetic mutants of NBCe1. Bolded text in the FWD primer shows the location of the codon 982 and 985 that were the subjects of mutagenesis.

Mutant	Title 2
AA	FWD: 5′-GAAGAAAGGA**GCT**TTGGAT**GCC**GACAATGACGATTCTG-3′ RVS: 5′-CAGAATCGTCATTGTCGGCATCCAAAGCTCCTTTCTTC-3′
AD	FWD: 5′-GAAGAAAGGA**GCT**TTGGAT**GAC**GACAATGACGATTCTG-3′ RVS: 5′-CAGAATCGTCATTGTCGTCATCCAAAGCTCCTTTCTTC-3′
DA	FWD: 5′-GAAGAAAGGA**GAT**TTGGAT**GCC**GACAATGACGATTCTG-3′ RVS: 5′-CAGAATCGTCATTGTCGGCATCCAAATCTCCTTTCTTC-3′
DD	FWD: 5′-GAAGAAAGGA**GAT**TTGGAT**GAC**GACAATGACGATTCTG-3′ RVS: 5′- CAGAATCGTCATTGTCGTCATCCAAATCTCCTTTCTTC-3′

## Data Availability

Data are available upon request to M.D.P.

## Supplementary Materials

The following are available online at https://www.mdpi.com/article/10.3390/ijms222312817/s1.
