# Supplementary material for: Revisiting the Role of Ser982 Phosphorylation in Stoichiometry Shift of the Electrogenic Na+/qHCO3− Cotransporter NBCe1
Source: Int J Mol Sci. 2021 Nov 26;22(23):12817. doi: 10.3390/ijms222312817 (PMC8657473; doi:10.3390/ijms222312817)
Supplement: Supplementary file 1 [file ijms-22-12817-s001.zip › ijms-1351084-supplementary.pdf]

Supplemental Figure S1 Western blot of mouse kidney protein probed with the anti-pSer982 antibody showing immunodepletion by pSer982 peptide (Pep2) as well as pS2r928/pSer985 peptide (Pep3)

Antigen: CKKKKG**pS**LDSD

Anti-pS982 antibody

Pep1 CKKKKG**S**LDSD
Pep2 CKKKKG**pS**LDSD
Pep3 KKKG**pS**LD**pS**DNDD

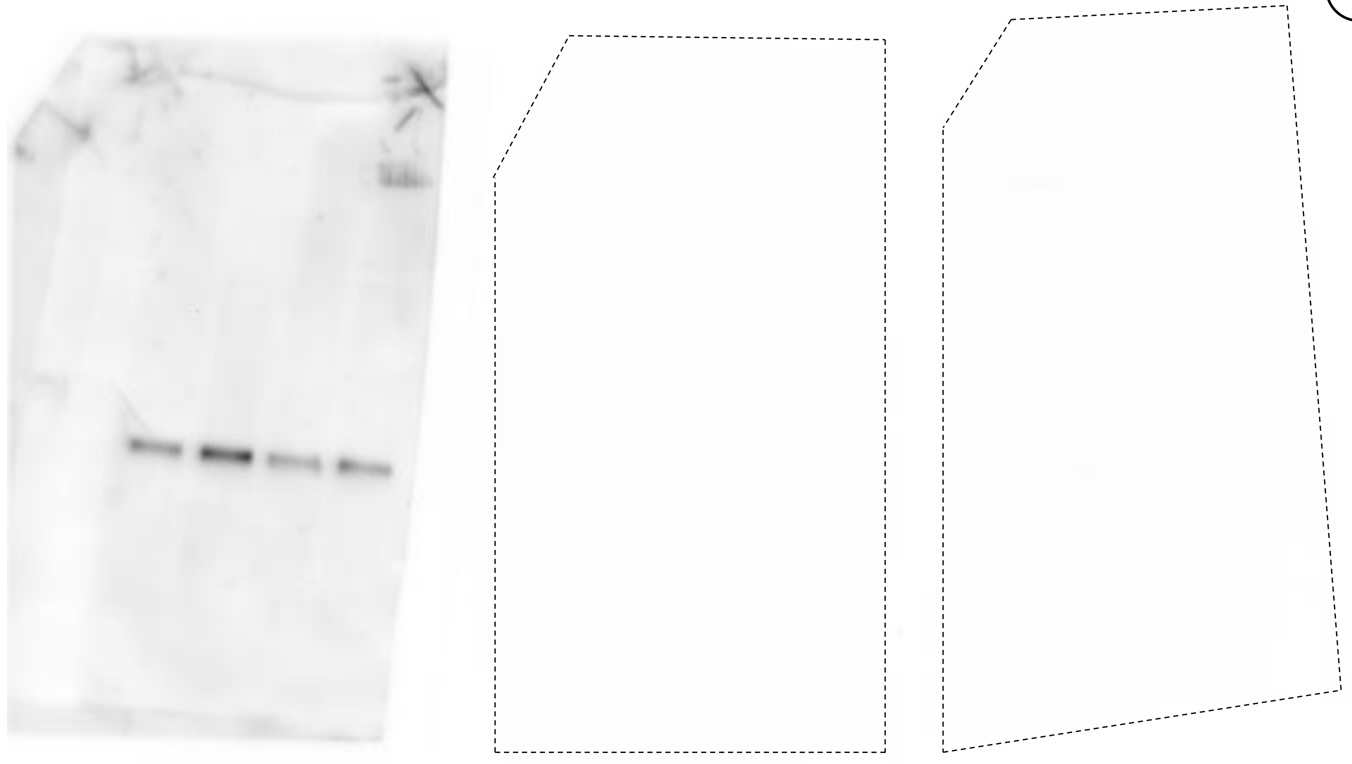

The three panels are triplicate blots from a single image. Dotted lines emphasize the outlines of the blots.

Preabsorption Peptide: Pep1 | Pep1 +Pep2 | Pep1 +Pep3

Supplemental Figure S2 Western blot of mouse colon protein probed with the anti-SLC4A4 antibody showing lack of NBCe1 immunoreactivity in the colons of NBCe1b/c-null mice

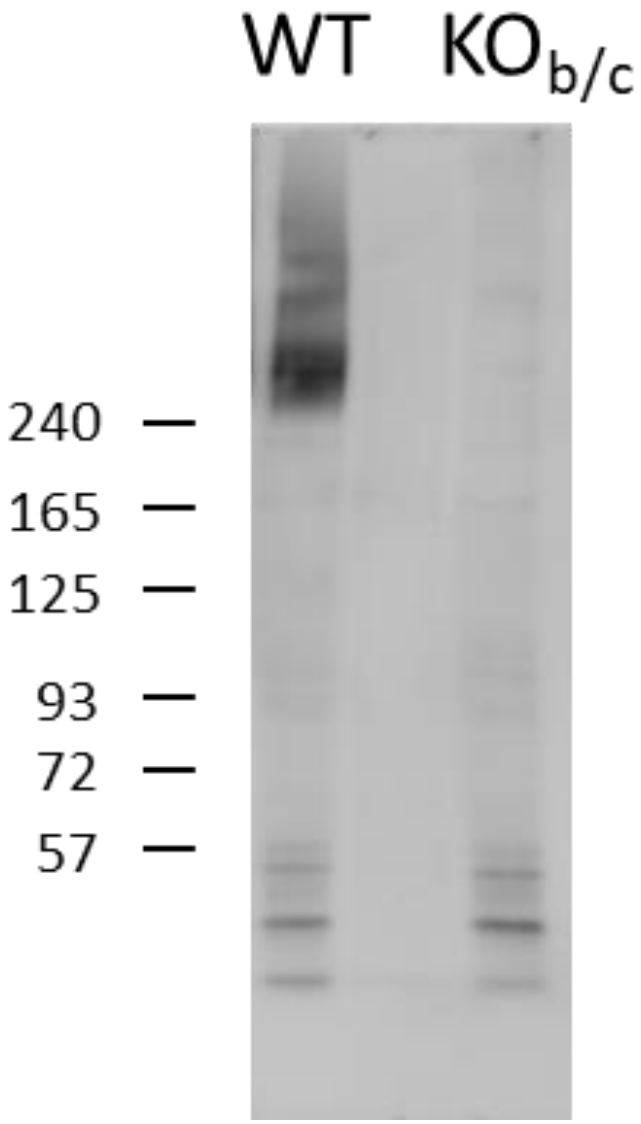

WT= wild-type mouse
$KO_{b/c}$= NBCe1b/c null mouse (Salerno et al., 2019)

Supplemental Figure S3 Total-protein stained PVDF membranes of western blots from control and acid-challenged groups

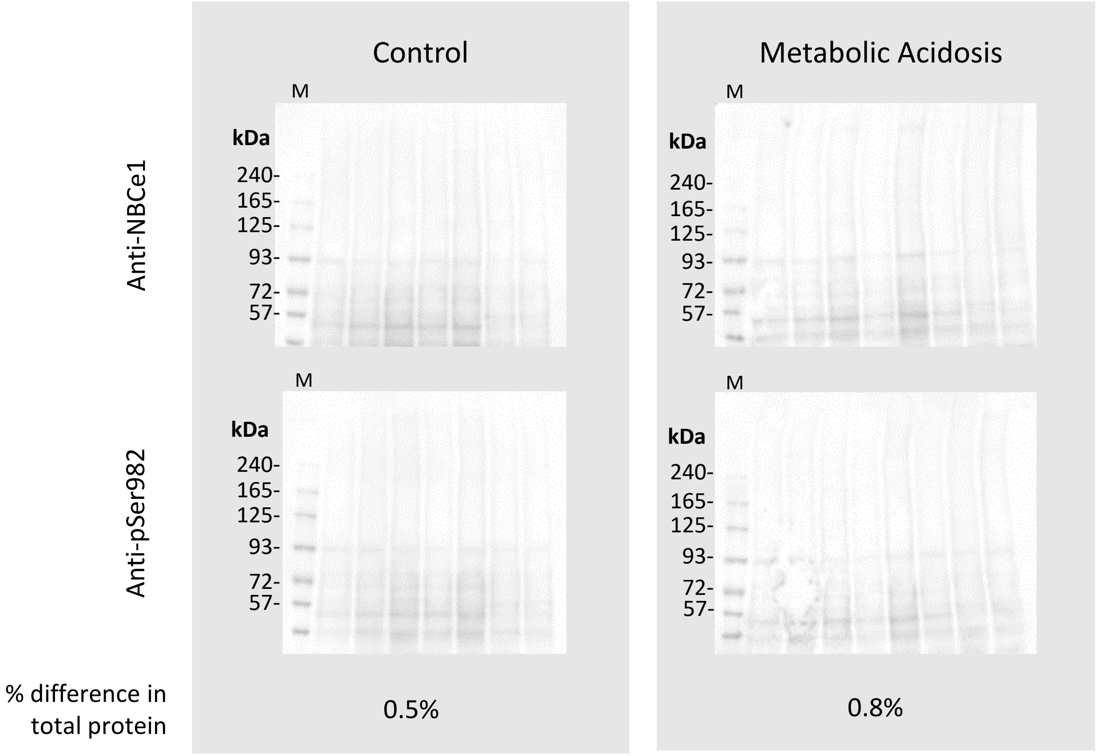

Western blots within experimental groups (Control and Acidosis) were identically loaded. To confirm equal transfer of protein, PVDF membranes were stained with the reversible Memcode total-protein stain, and the average intensity of the entire blot was assessed with Fiji. The percent difference in average intensity was calculated between the two “Control” blots, and the two “Metabolic Acidosis” blots. M= marker lane.
